# Integrating Mendelian randomization with single-cell and spatial transcriptomics to investigate gut microbiota-associated genetic signals and molecular characteristics in prostate cancer

**DOI:** 10.3389/fcimb.2026.1899354

**Published:** 2026-10-06

**Authors:** Chenjie Qiu, Huilin Zhang, Zongmin Du, Yuan Gao, Liangliang Dai

**Affiliations:** 1 Department of General Surgery, Changzhou Hospital of Traditional Chinese Medicine, Changzhou, China; 2 Department of Neurosurgery, Jiangmen Central Hospital, Jiangmen, China; 3 Department of Urology, Wujin Hospital Affiliated With Jiangsu University, Changzhou, China; 4 Department of Urology, The Wujin Clinical College of Xuzhou Medical University, Changzhou, China; 5 Department of Otolaryngology, Changzhou Medical Centre of the 904th Hospital, Chinese Logistics Support Force, Changzhou, China

**Keywords:** gut microbiota, Mendelian randomization, prostate cancer, single-cell transcriptomics, spatial transcriptomics

## Abstract

**Background:**

Prostate cancer (PRAD) represents one of the most prevalent malignancies among men globally. Accumulating evidence implicates gut microbiota dysbiosis in the initiation and progression of prostate cancer; however, the causal relationship and underlying molecular mechanisms remain incompletely elucidated. This study integrated Mendelian randomization (MR), bioinformatics, single-cell RNA sequencing, and spatial transcriptomics to systematically investigate the genetic associations between gut microbiota and prostate cancer risk and characterize the expression patterns of microbiota-associated candidate genes within the tumor microenvironment.

**Methods:**

Two-sample MR analyses were performed using summary statistics from the OpenGWAS database to evaluate causal associations between gut microbiota and PRAD risk, with sensitivity analyses conducted to ensure robustness. Significant SNPs were functionally annotated and mapped to candidate genes using g:SNPense, and differentially expressed associated genes (DEAGs) were identified through integration with GEO datasets. Subsequent analyses encompassed immune infiltration profiling, machine learning model construction, and nomogram validation. Single-cell RNA sequencing and spatial transcriptomics were employed to delineate cell type-specific expression and spatial distribution patterns of signature genes. Candidate gene expression alterations in prostate cancer cells were experimentally validated by quantitative real-time PCR (qRT-PCR).

**Results:**

MR analyses identified significant positive causal associations between PRAD risk and Brevibacillaceae, Brevibacillales, CAG-110, and Pararhizobium, whereas Lawsonibacter sp000492175 exhibited a significant inverse association. A total of 21 differentially expressed genes were identified, with RASSF3, CROCC, UBE4B, SMOC2, and ACACA emerging as key signature genes. Immune infiltration analyses revealed a significant positive correlation between FRMD3 and regulatory T cells (Tregs), and a negative correlation between ACACA and neutrophil infiltration. The random forest classifier demonstrated optimal predictive performance, achieving an area under the curve (AUC) of 0.853 in the validation cohort. Single-cell and spatial transcriptomic analyses revealed cell-type-associated expression patterns of ACACA, SMOC2, and UBE4B across different cellular populations. These findings suggest potential associations between signature genes and specific cellular contexts, which require further experimental validation. qRT-PCR validated differential expression patterns of these candidate genes in prostate cancer cells.

**Conclusion:**

This study provides genetic causal inference evidence supporting a close association between gut microbiota and prostate cancer risk, and identifies multiple key signature genes implicated in immune microenvironment regulation and tumor progression. Integrated single-cell and spatial transcriptomic analyses suggest that these genes exhibit distinct expression patterns among different cellular populations and may provide clues for understanding their potential roles in PRAD progression. These findings provide genetic evidence supporting the association between gut microbiota and prostate cancer risk and highlight potential microbiota-associated molecular signatures. Further functional studies are required to clarify whether these genes directly contribute to microbiota-related prostate cancer progression.

## 1 Introduction

Prostate cancer (PCa) is a malignant neoplasm arising from the aberrant proliferation of prostatic epithelial cells (Adamaki and Zoumpourlis, 2021). According to the 2022 global cancer statistics, PCa represents the second most commonly diagnosed malignancy and the fifth leading cause of cancer-related mortality among men worldwide, accounting for 14.2% of all new cancer cases and 7.3% of cancer-related deaths in males (Bray et al., 2024). Emerging evidence suggests that gut microbiota dysbiosis contributes to the progression of prostate cancer (Xu et al., 2020). Notably, Liss et al. (2018) reported that the fecal microbiota of patients with metastatic castration-resistant prostate cancer (CRPC) exhibited enhanced metabolic activities associated with steroid biosynthesis compared with individuals diagnosed with benign prostatic hyperplasia (BPH).

Accumulating evidence has implicated the microbiome in PCa development (Ohadian Moghadam and Momeni, 2021), with the gut microbiota being particularly closely associated with the initiation and progression of PCa. A growing body of literature has demonstrated that gut microbiota can modulate PCa risk, therapeutic response, and prognosis through multiple mechanisms (Katongole et al., 2020). In recent years, scientific understanding of human commensal microorganisms has advanced substantially, and the relationship between gut microbiota and various diseases has attracted considerable research attention. Gut microbiota dysbiosis can facilitate tumorigenesis through diverse pathways, including the induction of chronic inflammation and the generation of deleterious metabolites. This causal relationship has been firmly established in colorectal cancer (Si et al., 2021) and hepatocellular carcinoma (Schwabe and Jobin, 2013; Katongole et al., 2020), in which lipopolysaccharide (LPS) and other microbial metabolites play critical roles in hepatocarcinogenesis. Given the unique anatomical proximity between the prostate and rectum, pathological alterations in these organs may exert reciprocal influences.

In the present study, we performed a two-sample Mendelian randomization (MR) analysis to evaluate the causal relationship between gut microbiota and prostate adenocarcinoma (PRAD) risk, with multiple sensitivity analyses conducted to ensure the robustness of the findings. Significant single nucleotide polymorphisms (SNPs) were functionally annotated and mapped to candidate genes using g:SNPense(https://biit.cs.ut.ee/gprofiler/snpense), and differentially expressed associated genes (DEAGs) were identified through integration with Gene Expression Omnibus (GEO) datasets. Immune infiltration and correlation analyses were subsequently performed. Machine learning algorithms were further employed to screen for signature genes and construct predictive models. Moreover, by integrating single-cell and spatial transcriptomic data, we elucidated the functional roles of signature genes in PRAD from the perspectives of cell-type specificity and spatial expression patterns, thereby providing deeper insights into the association between gut microbiota-related genetic signals and prostate cancer molecular characteristics. Ultimately, this study aimed to explore the potential of intestinal microorganisms as novel biomarkers for predicting PCa.

## 2 Methods

### 2.1 Collection of GWAS and GEO datasets

Genetic instrumental variables for gut microbiota exposure and outcome data were derived from recently published genome-wide association studies (GWAS) of the gut microbiome available through the IEU OpenGWAS database (https://opengwas.io/). Fecal sample-based GWAS quantified the abundance of 473 taxa, including species-level analyses. These data were generated by Qin et al. (2024) from metagenomic sequencing of 5,959 European individuals enrolled in the Finnish cohort (FR02), identifying 473 taxa classified according to the Genome Taxonomy Database (GTDB), representing 17% of all tested taxa across 11 phyla, 19 classes, 24 orders, 62 families, 146 genera, and 209 species. Given the relatively modest sample size of the gut microbiome GWAS (n = 5,959), applying the conventional genome-wide significance threshold of P < 5 × 10⁻^8^ would substantially reduce the number of available instrumental variables for some microbial taxa and consequently limit the statistical power of the MR analysis. Therefore, a relaxed significance threshold of P < 1 × 10⁻^6^ was adopted for SNP selection (Liu et al., 2024; Dong et al., 2025). To ensure independence among instrumental variables, LD clumping was performed using a threshold of r² < 0.001 within a 10-Mb window.

The strength of each instrumental variable was further evaluated by calculating the proportion of variance in the exposure explained by each SNP (R²) and the corresponding F-statistic. For each SNP, R² was calculated as:


$$R^{2}=\frac{2\beta^{2}\times EAF\times \left(1-EAF\right)}{2\beta^{2}\times EAF\times \left(1-EAF\right)+2SE^{2}\times N\times EAF\times \left(1-EAF\right)}$$

where β represents the estimated SNP–exposure effect, SE represents the corresponding standard error, EAF denotes the effect allele frequency, and N represents the sample size of the exposure GWAS. Effect allele frequencies were obtained from the European population of the 1000 Genomes Project Phase 3 and aligned according to the effect allele. The F-statistic for each SNP was subsequently calculated as:


$$F=\frac{R^{2}\left(N-2\right)}{1-R^{2}}$$

SNPs with F-statistics > 10 were considered sufficiently strong instrumental variables and were retained for subsequent MR analyses, whereas SNPs with F-statistics ≤ 10 were excluded to minimize potential weak-instrument bias.

Raw GWAS summary statistics are publicly accessible from the NHGRI-EBI GWAS Catalog (https://www.ebi.ac.uk/gwas/, accession numbers GCST90032172–GCST90032644) and the IEU OpenGWAS database (https://gwas.mrcieu.ac.uk/) (**Supplementary Material 1**). Outcome data for PRAD were obtained from the GWAS dataset “ieu-b-85” (**Supplementary Material 2**).

Transcriptomic data were retrieved from the Gene Expression Omnibus (GEO) database (https://www.ncbi.nlm.nih.gov/geo/) using the search term “prostate cancer,” yielding three datasets: GSE46602, GSE73397, and GSE8511 (**Supplementary Material 3**). All datasets were derived from human prostatic tissue specimens. The first two datasets served as training cohorts, comprising 39 PRAD patient samples and 17 control samples. The third dataset, consisting of 25 PRAD samples and 16 control samples, was designated as the validation cohort. Following processing and integration, a comprehensive expression matrix was constructed, encompassing gene expression levels for both control and PRAD groups. Single-cell data were obtained from GEO by searching for “prostate cancer single-cell sequencing,” yielding dataset GSE278936 (**Supplementary Material 4**), which included 52 prostate tumor specimens: 4 benign prostatic hyperplasia tissue samples, 4 metastatic prostate cancer patient tissue samples, and 44 primary prostate cancer tissue samples.

### 2.2 Mendelian randomization analysis

In the first step of this study, a two-sample MR analysis was conducted, utilizing SNPs as instrumental variables to investigate the causal relationship between gut microbiota and PRAD risk (Verduijn et al., 2010; Burgess et al., 2017). To ensure valid causal inference, MR analysis requires that the instrumental variables satisfy the following assumptions: (i) the SNPs are strongly associated with the exposure; (ii) the SNPs are independent of confounding factors; and (iii) the SNPs influence the outcome exclusively through the exposure. This study adhered to the STROBE-MR (Strengthening the Reporting of Observational Studies in Epidemiology using Mendelian Randomization) checklist (Liu et al., 2025).

We performed two-sample MR analyses using the TwoSampleMR package to examine causal associations between various gut microbiota taxa and PRAD. The inverse variance weighted (IVW) method under a random-effects model served as the primary analytical approach, given its precision and reliability in MR analyses. Because 473 gut microbial taxa were evaluated as exposure variables, multiple testing was considered when interpreting MR results. In this study, the MR analysis was designed as an exploratory screening strategy to identify candidate microbiota taxa for subsequent functional characterization rather than a definitive hypothesis-testing framework. Therefore, nominal IVW P values were used for candidate prioritization. Although Bonferroni correction would provide a conservative control of false-positive findings (threshold: 0.05/473 = 1.06 × 10⁻^4^), such correction may substantially reduce statistical power in microbiome-wide MR analyses with relatively limited exposure GWAS sample sizes and may eliminate potentially informative taxa for downstream biological investigation. Therefore, the identified taxa were considered candidate associations requiring further validation through genetic annotation, transcriptomic integration, machine learning, and single-cell/spatial transcriptomic analyses. The identified microbiota taxa were subsequently investigated through SNP annotation, transcriptomic analysis, machine learning, and single-cell/spatial transcriptomic analyses to provide additional biological support. Associations identified in the MR analysis were further evaluated based on both statistical significance and biological plausibility. When only one SNP was available as an instrumental variable for an exposure, the Wald ratio method was applied. To evaluate the robustness of the MR findings, several statistical tests were employed. Cochran’s Q statistic was used to assess heterogeneity, with P < 0.05 indicating the presence of heterogeneity. Horizontal pleiotropy was evaluated using MR-Egger regression and MR Pleiotropy Residual Sum and Outlier (MR-PRESSO), with P < 0.05 suggesting the presence of horizontal pleiotropy (Huang et al., 2021). To further enhance result credibility, linkage disequilibrium score regression (LDSC) was employed as a complementary approach to calculate genetic correlations between gut microbiota taxa and PRAD.

### 2.3 SNP-to-gene mapping

Following the two-sample MR analysis, SNPs associated with gut microbiota taxa showing significant causal associations with PRAD were subjected to functional annotation and candidate gene prioritization. The g:SNPense(https://biit.cs.ut.ee/gprofiler/snpense) tool was used to annotate these SNPs by identifying their corresponding gene names, chromosomal locations, and variant consequence terms according to the Sequence Ontology. The expression profiles of annotated genes were subsequently extracted from merged GEO datasets to identify candidate genes for downstream differential expression, machine learning, single-cell, and spatial transcriptomic analyses. Importantly, this SNP-to-gene mapping approach was used for functional prioritization of candidate genes and does not represent a formal statistical colocalization analysis.

### 2.4 Identification and analysis of candidate genes

Subsequently, PRAD datasets (partitioned into training and validation cohorts) were obtained from the merged GEO database (Barrett et al., 2009), encompassing both control and PRAD groups. Differential expression analysis of candidate genes was performed between groups. Genes with P < 0.05 were defined as differentially expressed associated genes (DEAGs), with results visualized using box plots. Chromosomal locations of DEAGs were displayed on a Circos plot. Furthermore, pairwise correlation coefficients between DEAGs were calculated using the cor command and visualized accordingly.

### 2.5 Immune cell infiltration analysis of candidate genes

Immune cell infiltration was assessed using CIBERSORT (Cell-type Identification By Estimating Relative Subsets Of RNA Transcripts) with 1,000 permutations, employing the reference file available at https://www.ncbi.nlm.nih.gov/pmc/articles/PMC4739640/bin/NIHMS670442-supplement-2.xlsx to obtain relative immune cell fractions summing to 1, which were displayed using bar plots. Subsequently, single-sample gene set enrichment analysis (ssGSEA) was performed to compare differences in immune cell composition between control and PRAD groups, with results presented as box plots. Correlation analyses between DEAGs and ssGSEA scores were conducted to obtain correlation coefficients, which were visualized as heatmaps.

### 2.6 Identification of signature genes and construction and validation of machine learning-based nomograms

Four predictive models were constructed using DEAG expression data: random forest (RF), support vector machine (SVM), generalized linear model (GLM), and extreme gradient boosting (XGBoost). The dataset was randomly divided into training and validation cohorts using stratified sampling. Model construction was performed using the training cohort, while the validation cohort was used for independent evaluation of predictive performance. To improve model stability and reduce the influence of sampling variation, repeated five-fold cross-validation was applied during model training. The candidate genes included in machine learning analyses were obtained from previous bioinformatics screening procedures, including differential expression analysis and biological relevance assessment. Therefore, machine learning algorithms were applied to compare the predictive capability of predefined candidate genes rather than performing unrestricted feature selection from the whole transcriptome dataset. The top five genes ranked by model importance scores were identified as signature genes. Nomograms were subsequently established based on the expression of signature genes in control and PRAD groups. To assess the accuracy and generalizability of the nomograms, decision and calibration curves were generated. Additionally, identical models were constructed using the validation cohort, and ROC curves were plotted to validate the training set models (Wu et al., 2025). The four machine learning algorithms were compared according to multiple evaluation criteria, including prediction error, ROC-AUC performance, and validation cohort results. Default parameter settings implemented in the caret package were used for model training, and no additional manual hyperparameter optimization was performed. Model performance was evaluated using repeated five-fold cross-validation to ensure robustness of the predictive results. The model showing the most robust overall predictive performance was selected for subsequent nomogram construction and validation.

### 2.7 Characterization of signature genes in PRAD through single-cell sequencing and spatial transcriptomics

In this study, single-cell transcriptomic sequencing was performed on PRAD and control groups. All single cells underwent cell clustering and type annotation. Expression profiles of signature genes were subsequently analyzed to elucidate their expression characteristics. The mean expression levels and positive cell proportions across cell subpopulations were quantified, with alluvial diagrams used to illustrate the correspondence between cell types and marker genes as well as their differential expression levels. Pseudotime trajectory analysis was performed to characterize continuous transcriptional state variation among cell populations. The inferred trajectories were interpreted as computational predictions of cellular state transitions rather than definitive biological differentiation pathways. Finally, spatial transcriptomic data were integrated to analyze the tissue spatial distribution of the three signature genes, clarifying their spatial expression characteristics across various cell populations.

### 2.8 Cell lines and cell culture

Human normal prostatic epithelial cells (RWPE-1) and human prostate cancer cells (VCaP and PC-3) were purchased from iCell Bioscience Inc. (China). All cell lines tested negative for mycoplasma contamination prior to experimentation. RWPE-1 cells were cultured in Keratinocyte-Serum Free Medium (KSFM, No. 10450-013, Gibco, MT, USA) supplemented with 5 ng/mL epidermal growth factor (No. 10744-019, Gibco). VCaP cells were maintained in high-glucose Dulbecco’s Modified Eagle Medium (DMEM) containing 10% fetal bovine serum (FBS, Gibco, USA) and 1% penicillin-streptomycin (Gibco, USA). PC-3 cells were cultured in F-12K medium supplemented with 10% FBS and 1% penicillin-streptomycin. All cells were incubated at 37 °C in a humidified atmosphere containing 5% CO_2_. Culture medium was replenished every 2–3 days according to cell growth status. Upon reaching 80%–90% confluence, cells were passaged using 0.25% Trypsin-EDTA.

### 2.9 Quantitative real-time PCR

Total RNA was extracted from RWPE-1, VCaP, and PC-3 cells using TRIzol reagent (Invitrogen, USA) according to the manufacturer’s instructions. Complementary DNA (cDNA) was subsequently synthesized using a reverse transcription kit (Vazyme Biotech, China). Quantitative real-time PCR (qRT-PCR) was performed to detect the mRNA expression levels of ACACA, CROCC, RASSF3, SMOC2, and UBE4B.

The qPCR reaction mixture had a total volume of 20 μL, comprising 10 μL 2× SYBR Premix, 1 μL cDNA template, 0.4 μL forward primer, 0.4 μL reverse primer, and 8.2 μL double-distilled water (ddH_2_O). The PCR amplification protocol was as follows: initial denaturation at 95 °C for 5 min; followed by 40 cycles of denaturation at 95 °C for 30 s and annealing at 60 °C for 30 s.

β-actin served as the internal reference gene, and the relative expression levels of target genes were calculated using the 2^−ΔΔCt method. All primers were synthesized by Sangon Biotech (China), and the primer sequences were as follows:

ACACA: 5′-ATGGGCGGAATGGTCTCTTTC-3′ (forward), 5′-TGGGGACCTTGTCTTCATCAT-3′ (reverse);
CROCC: 5′-GGTGGAGCTGACACTAGAGAC-3′ (forward), 5′-GGCTGGTGATCTGAGCGTC-3′ (reverse);
RASSF3: 5′-TCAAATGGGATTTACACTGGCTTC-3′ (forward), 5′-GCTTGCAGGCGTAGACTTGG-3′ (reverse);
SMOC2: 5′-GTCGGCAGAACAAAACCAAT-3′ (forward), 5′-CACCACATTGTCGTTCTTGG-3′ (reverse);
UBE4B: 5′-TCCTTCCTCGTGCCGTACAT-3′ (forward), 5′-GTGGTCCTCACCAGCTCTTGA-3′ (reverse);
β-actin: 5′-CACCATTGGCAATGAGCGGTTC-3′ (forward), 5′-AGGTCTTTGCGGATGTCCACGT-3′ (reverse).

## 3 Statistical analysis

All statistical analyses were performed using R software (version 4.4.2). For MR analyses involving 473 gut microbial taxa, the potential influence of multiple testing was considered. Nominal P values from the inverse variance weighted (IVW) method were used for exploratory candidate identification, as the MR analysis was designed as a hypothesis-generating screening strategy rather than a definitive hypothesis-testing framework. Given the large number of microbial exposures evaluated, potential false-positive findings due to multiple testing were considered as an important limitation of this study. Although no formal false discovery rate (FDR) correction was applied in the primary MR screening, the identified associations were further evaluated through sensitivity analyses and integrated multi-omics analyses to assess their biological plausibility. Comparisons between two independent groups were conducted using the independent samples t-test, whereas paired samples were analyzed using the Wilcoxon signed-rank test. For comparisons among three or more groups, one-way analysis of variance (ANOVA) or the Kruskal–Wallis rank-sum test was employed, as appropriate. Correlation analyses were performed using Spearman’s rank correlation test. For other statistical analyses, including transcriptomic comparisons and correlation analyses, Benjamini–Hochberg adjustment was applied where appropriate, and an adjusted P-value or false discovery rate (FDR) <0.05 was considered statistically significant.

## 4 Results

### 4.1 Mendelian randomization analysis

MR analysis identified potential genetically informed associations between gut microbiota taxa and prostate cancer (PRAD) risk under an exploratory screening framework. All instrumental variables included in the MR analyses showed adequate instrument strength, with F-statistics ranging from approximately 19 to 31, all exceeding the conventional threshold of 10. These findings indicate that weak-instrument bias was unlikely to substantially affect the MR estimates. Detailed F-statistics for individual SNPs are provided in the **Supplementary Material**. Among the 473 gut microbial taxa analyzed, five taxa showed nominally significant associations with PRAD risk based on IVW analysis and were selected as candidate microbiota for subsequent functional analyses. Considering the exploratory nature of the MR screening and the limited statistical power of microbiome-wide analyses, these nominal associations were further evaluated through sensitivity analyses, SNP annotation, transcriptomic integration, machine learning, and single-cell/spatial transcriptomic analyses. Brevibacillaceae was positively associated with PRAD risk (IVW OR = 1.631, 95% CI: 1.259–2.113, nominal P < 0.001). With approximately 15 SNPs included, the effect sizes in the forest plot were predominantly distributed in the positive range; the funnel plot was symmetrical; the leave-one-out analysis was stable; and the regression lines in the scatter plots all exhibited a positive slope (**Figures 1A–D**). Concurrently, Brevibacillales was also significantly positively correlated with PRAD risk (OR = 1.690, 95% CI: 1.311–2.179, P < 0.001), with approximately 17 SNPs included. No significant bias was observed in the sensitivity analyses, and the results were robust (**Figures 1E–H**). Furthermore, the abundance of CAG-110 was found to be moderately but significantly positively correlated with PRAD risk (OR = 1.117, 95% CI: 1.031–1.210, P = 0.007), with approximately 13 SNPs included; although individual effects showed some variation, the overall trend remained stable (**Figures 1I–L**). In contrast, the abundance of Lawsonibacter sp000492175 was significantly negatively correlated with PRAD risk (OR = 0.685, 95% CI: 0.552–0.849, P < 0.001). With nine SNPs included, the forest plot effects were concentrated in the negative range, and the leave-one-out analysis consistently showed a stable negative trend, suggesting a protective effect (**Figures 1M–P**). Meanwhile, the abundance of Pararhizobium was significantly positively correlated with PRAD risk (OR = 1.401, 95% CI: 1.134–1.731, P = 0.002), with a total of 19 SNPs included; the effect estimates were concentrated and consistent (**Figures 1Q–T**). The number of SNPs for the five bacterial groups ranged from 9 to 19, all meeting the criteria for MR analysis. Among these, Brevibacillaceae, Brevibacillales, CAG-110 and Pararhizobium were risk-associated bacterial groups, whilst sp000492175 was a protective bacterial group (**Figure 1U**).

**Figure 1:**
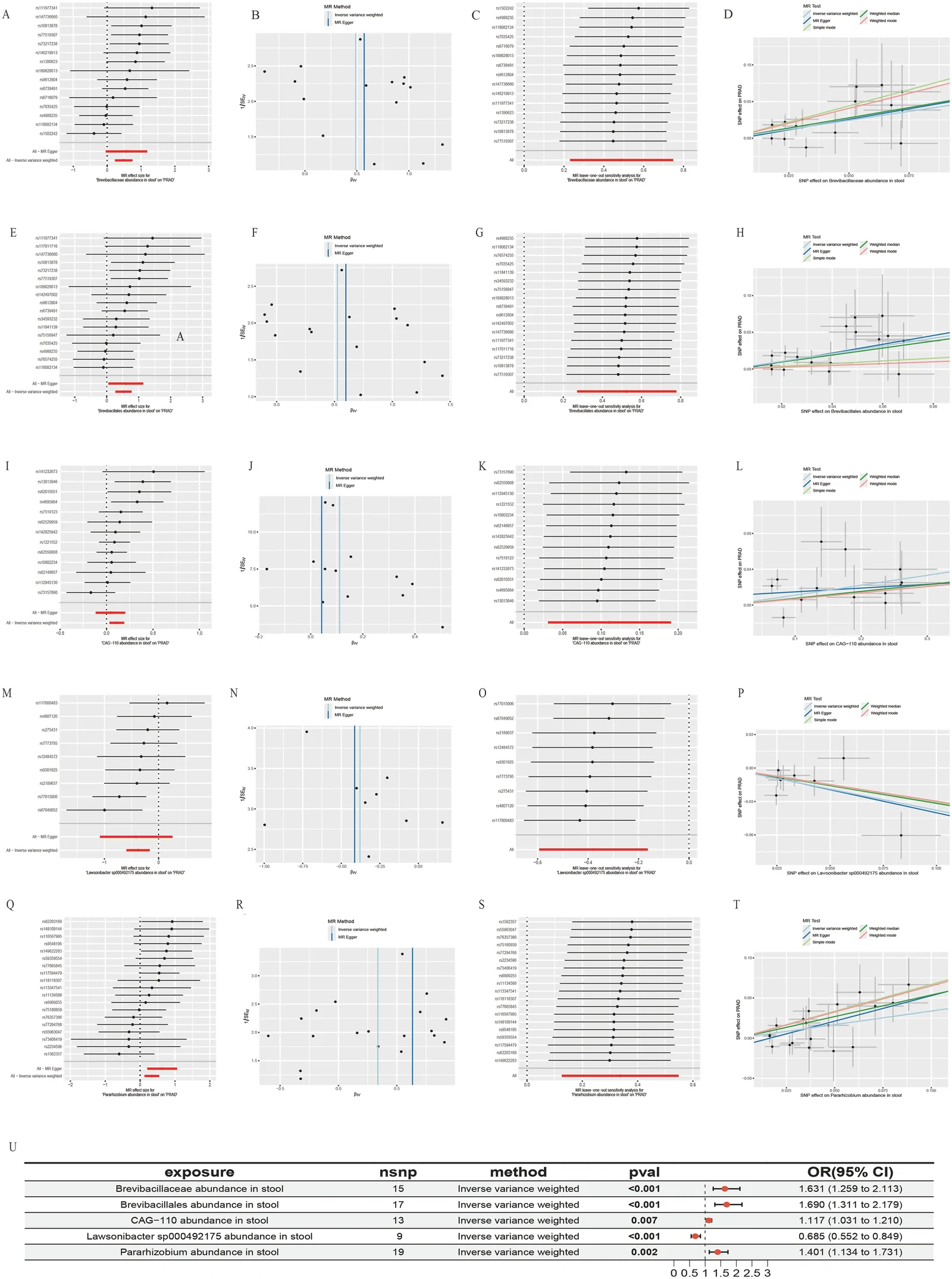
Gut microbiota and MR Of prostate cancer. **(A–D)** MR analysis of Brevibacillaceae abundance and prostate cancer risk, including scatter plot, forest plot, funnel plot, and leave-one-out sensitivity analysis. **(E–H)** MR analysis of Brevibacillales abundance and prostate cancer risk, including scatter plot, forest plot, funnel plot, and leave-one-out sensitivity analysis. **(I–L)** MR analysis of CAG-110 abundance and prostate cancer risk, including scatter plot, forest plot, funnel plot, and leave-one-out sensitivity analysis. **(M–P)** MR analysis of Lawsonibacter sp000492175 abundance and prostate cancer risk, including scatter plot, forest plot, funnel plot, and leave-one-out sensitivity analysis. **(Q–T)** MR analysis of Pararhizobium abundance and prostate cancer risk, including scatter plot, forest plot, funnel plot, and leave-one-out sensitivity analysis. **(U)** Forest plot of candidate gut microbiota taxa identified by exploratory MR analysis using the IVW method, showing the causal effects of each taxon on prostate cancer risk (OR and 95% CI). [The P values shown in the figure represent nominal IVW P values used for exploratory candidate screening. No multiple-testing-adjusted P values were applied in the exploratory MR screening, and these taxa were further investigated using downstream functional analyses].

### 4.2 Differential expression analysis of candidate genes

MR analysis identified 73 SNPs associated with microbiota-related causal effects on prostate cancer risk, which were mapped to 23 candidate genes for further functional prioritization. Following differential expression analysis against the PRAD GEO dataset, 21 differentially expressed genes were identified (**Figure 2A**). Among these, FRMD3, UBE4B, RASSF3, CROCC, SMOC2 and ACACA exhibited significant intergroup expression differences in the clustering heatmap (**Figure 2B**). Chromosomal localisation analysis revealed that these core genes are distributed across different chromosomal regions, with UBE4B and CROCC localised in adjacent regions on chromosome 1 (**Figure 2C**). Correlation analysis further indicated that RASSF3 is positively correlated with ACACA, whilst SMOC2 is negatively correlated with ACACA (**Figure 2D**).

**Figure 2:**
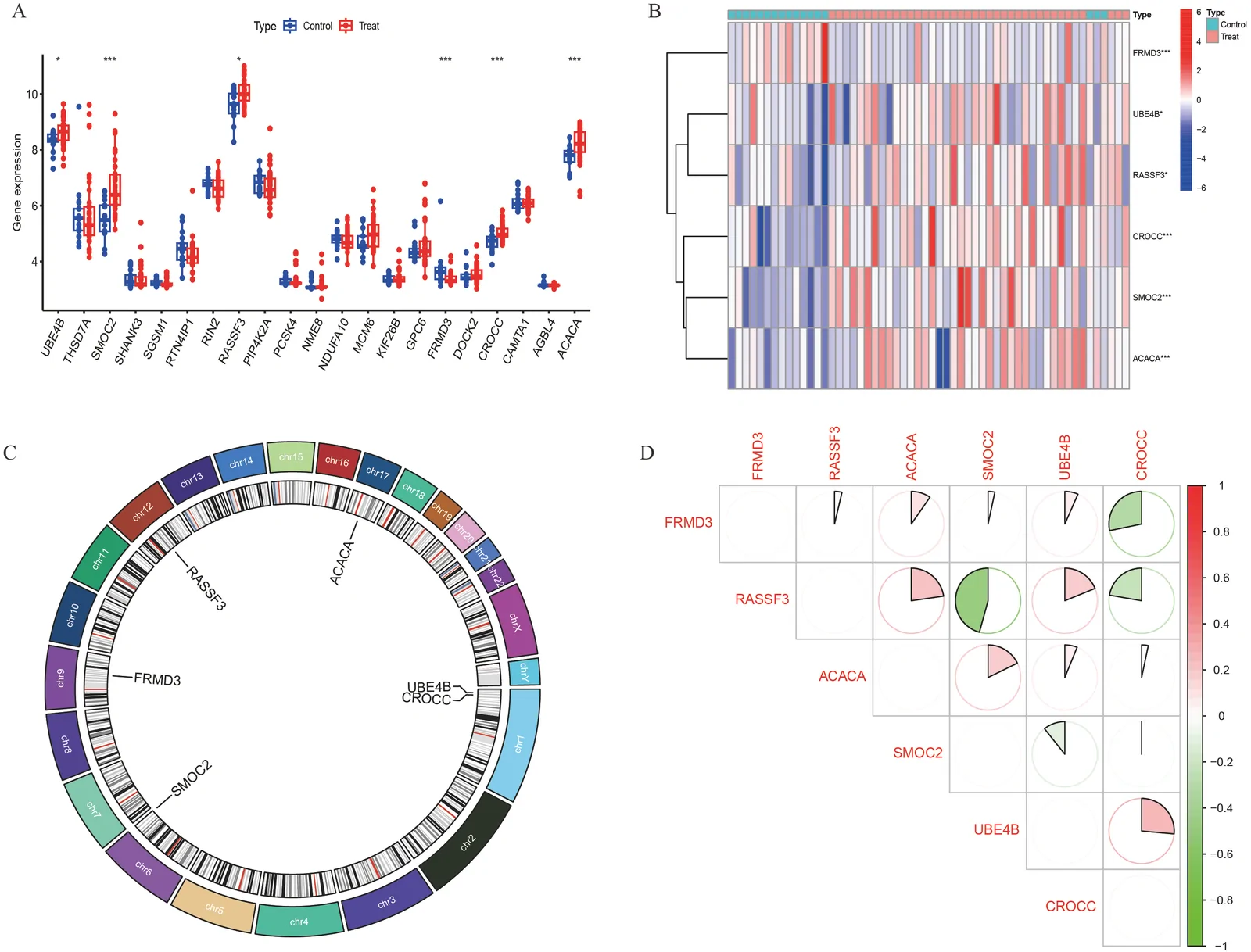
Identification and expression signature analysis of core differentially expressed genes. **(A)** Box plot of differential expression analysis of 21 genes in Control and Treat groups. **(B)** Unsupervised hierarchical clustering heatmap of mRNA expression levels of the six core differentially expressed genes. **(C)** Chromosomal ring mapping of the six core differentially expressed genes. **(D)** Matrix diagram for co-expression correlation analysis of the six core differentially expressed genes.

### 4.3 Immune cell correlation analysis

Analysis of immune infiltration profiles using the CIBERSORT algorithm (**Figure 3A**) revealed significant differences between the Treat and Control groups in the proportions of 22 immune cell types, with statistically significant differences observed between the two groups for subpopulations such as CD4-naive T cells, follicular helper T cells and regulatory T cells (Tregs) (**Figure 3B**). Further correlation analysis revealed (**Figure 3C**) that FRMD3 was significantly positively correlated with Tregs, whilst ACACA was negatively correlated with innate immune cells such as neutrophils. This suggests that the treatment may reshape the tumour immune microenvironment by regulating key genes such as FRMD3 and ACACA, thereby influencing the differentiation of T cell subsets and the level of neutrophil infiltration.

**Figure 3:**
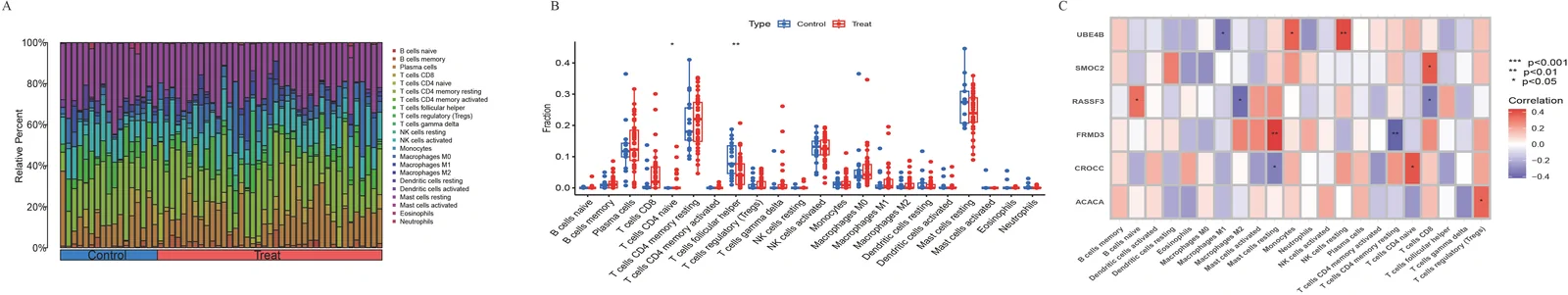
Immune cell infiltration analysis and core gene-immune cell association analysis. **(A)** Stacked bar chart of immune cell infiltration in Control and Treat groups. **(B)** Box plot of differential infiltration analysis of 22 immune cell subsets between control and treated groups. **(C)** Heat map of correlation analysis between six core differential genes and 22 immune cell subsets.

### 4.4 Machine learning model selection, nomogram construction, and validation

In this study, generalized linear model (GLM), support vector machine (SVM), random forest (RF), and extreme gradient boosting (XGB) models were constructed based on candidate differentially expressed genes, and their performance was systematically compared. Comparative evaluation of the four machine learning algorithms showed that the RF model exhibited relatively stable predictive performance, with lower prediction error and improved discrimination ability compared with SVM, GLM, and XGBoost (**Figures 4A–C**). Feature importance analysis was performed for the four machine learning models (**Figure 4B**). ROC analysis further supported the diagnostic capability of the RF model, which achieved a higher AUC than SVM, GLM, and XGBoost (**Figure 4D**). Considering the integrated evaluation of prediction error, discrimination ability, and validation cohort performance, RF was selected for subsequent nomogram construction. A nomogram prediction model was constructed using the feature genes identified via machine learning (**Figure 4G**). The calibration curve demonstrated good agreement between the model’s predicted probability and the actual probability of occurrence (**Figure 4E**), whilst decision curve analysis (DCA) further confirmed that the model offers good clinical net benefit across a wide range of threshold probabilities (**Figure 4F**). Subsequent external validation of the nomogram model revealed that the model possesses good discriminatory ability, with an AUC of 0.853 (95% CI: 0.730–0.948) (**Figure 4H**). Further evaluation of the diagnostic value of individual genes revealed that ACACA exhibited the highest diagnostic performance (AUC = 0.760), followed by CROCC (AUC = 0.688), UBE4B (AUC = 0.628), SMOC2 (AUC = 0.620) and FRMD3 (AUC = 0.570) (**Figure 4I**), suggesting that the multigene combined model exhibits superior diagnostic performance compared to single-gene models. The final external validation results demonstrated that the model maintained relatively stable diagnostic performance (**Figure 4J**).

**Figure 4:**
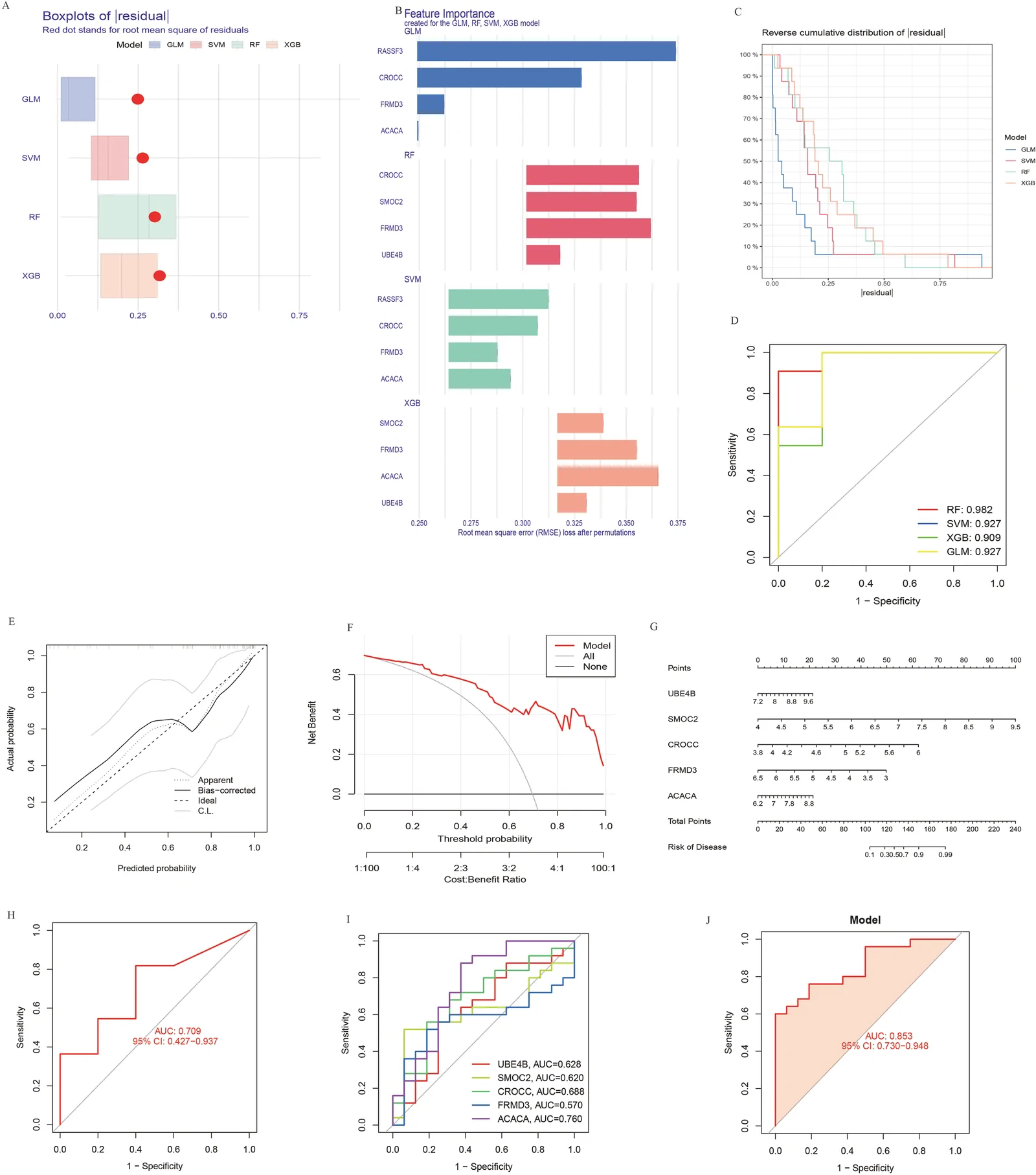
Machine learning model construction, performance evaluation and validation **(A)** Box plots of absolute residuals for the four machine learning models, including generalized linear model (GLM), support vector machine (SVM), random forest (RF), and extreme gradient boosting (XGB). **(B)** Feature importance analysis for the four machine learning models. **(C)** Inverse cumulative distribution curves of absolute residuals for GLM, SVM, RF, and XGB. **(D)** ROC curves comparing the predictive performance of GLM, SVM, RF, and XGB. **(E)** Model calibration curves. **(F)** Decision curve analysis (DCA). **(G)** Risk-score nomogram based on the core genes. **(H)** ROC curve of the multigene model in the training cohort. **(I)** ROC curves for the five core genes. **(J)** ROC curve of the multigene model in the external GEO validation cohort.

### 4.5 Single-cell sequencing and spatial transcription

The results of single-cell sequencing analysis revealed that, following dimensionality reduction and clustering, multiple cell subpopulations were identified and classified into major cell types—including epithelial cells, fibroblasts, smooth muscle cells, tissue stem cells and iPS cells—through cell annotation (**Figures 5A, B**). Different cell populations exhibited distinct separation in the UMAP space, indicating significant cellular heterogeneity (**Figure 5A**). Subsequent single-cell expression profiling of candidate genes revealed that CROCC, FRMD3, UBE4B, SMOC2 and ACACA all exhibited cell-type-specific expression patterns across different cell subpopulations, with the majority of these genes predominantly distributed within subpopulations associated with tissue stem cells (**Figures 5C–G**). Pseudotime analysis demonstrated continuous variation of transcriptional states among cells. These computational trajectories may reflect potential cellular state transitions but should not be interpreted as definitive developmental lineages (**Figure 5H**). Concurrently, CROCC, FRMD3 and UBE4B showed expression variation along the inferred pseudotime ordering, suggesting differences in transcriptional states among cellular populations (**Figures 5I–K**). Spatial transcriptomic analysis further annotated the cellular composition within the tissue, revealing distinct spatial distribution patterns for tissue stem cells, smooth muscle cells, epithelial cells, fibroblasts and endothelial cells (**Figure 5L**). Analysis of marker genes revealed distinct expression profiles across different cell types, enabling the identification of cell-specific marker genes for each population (**Figure 5M**). Dot plot analysis further revealed significant differences in expression levels and the proportion of positive cells for ACACA, CROCC, FRMD3, SMOC2 and UBE4B across different cell subpopulations (**Figure 5N**). Furthermore, pseudo-temporal expression analysis indicated that ACACA and SMOC2 exhibit dynamic expression patterns during cell development (**Figure 5O**).

**Figure 5:**
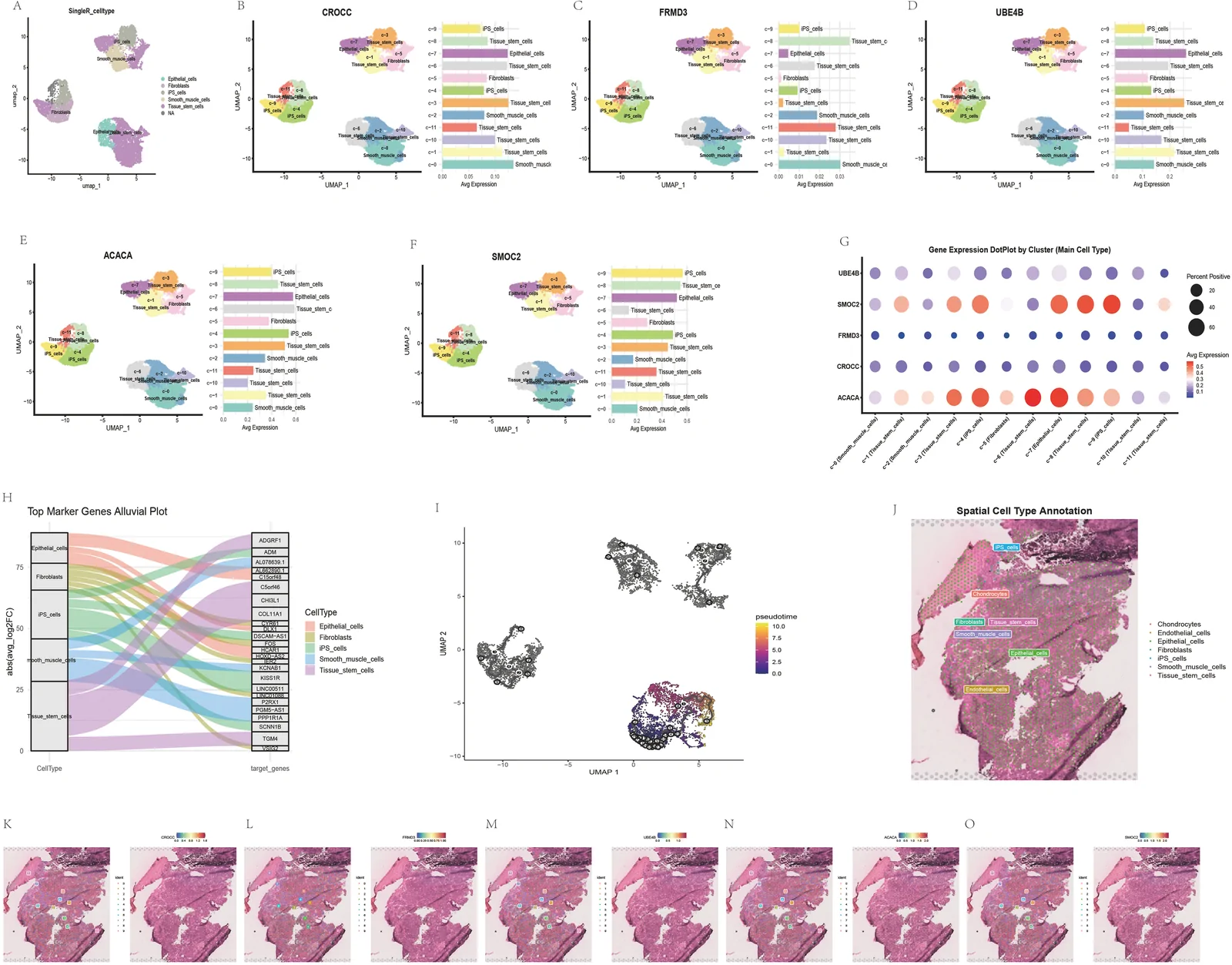
Single-cell sequencing and spatial transcription **(A)** UMAP dimensionality reduction graph of cell types annotated by SingleR algorithm. **(B–F)** The expression distribution of five target genes (UBE4B, SMOC2, FRMD3, CROCC, ACACA) in the single-cell UMAP space (left) and the average expression of each cell subset (cluster c-0 to c-11) bar graph (right). Shades of colour represent gene expression levels, and bars mark the major cell types corresponding to each cluster. **(G)** Bubble plot (DotPlot) of the expression of five target genes (UBE4B, SMOC2, FRMD3, CROCC, ACACA) in each cell subpopulation. Bubble size indicates the percentage of cells expressing the gene (Percent Positive), and colour depth indicates the average Expression (Avg Expression). **(H)** Alluvial Plot of Top marker genes for each cell type. The left axis is the cell type, the right axis is the corresponding marker gene, the line width reflects the strength of association between the gene and the cell type, and the colour corresponds to different cell types. **(I)** UMAP map of single cell Pseudotime analysis. Colours ranging from dark blue (0.0) to yellow (10.0) indicate inferred pseudotime values representing the relative ordering of cells based on transcriptional similarity. **(J)** Cell-type annotation map on spatial transcriptome tissue sections. The spatial localization of seven cell types: Chondrocytes, Endothelial_cells, epithelial cells, fibroblasts, iPS cells, smooth muscle cells and tissue stem cells were superimposed on the H&E staining background. **(K–O)** Expression profiles of five target genes (UBE4B, SMOC2, FRMD3, CROCC, ACACA) on spatial transcriptome tissue sections. The colour gradient indicates the expression intensity of the gene in different spatial regions.

### 4.6 Detection of the expression levels of ACACA, CROCC, RASSF3, SMOC2 and UBE4B in prostate cancer cells

To investigate changes in the expression of candidate genes in prostate cancer, this study employed qRT-PCR to detect the mRNA expression levels of ACACA, CROCC, RASSF3, SMOC2 and UBE4B in normal prostate epithelial cells (RWPE-1) and prostate cancer cells (VCaP and PC-3). The results showed that, compared with RWPE-1 cells, the mRNA expression level of ACACA was significantly elevated in VCaP cells (P < 0.001), whereas the expression of ACACA in PC-3 cells did not differ statistically significantly from that in RWPE-1 cells (P > 0.05) (**Figure 6A**). CROCC expression was significantly higher in both VCaP and PC-3 cells than in RWPE-1 cells, with the increase being more pronounced in PC-3 cells (all P < 0.001) (**Figure 6B**). RASSF3 was significantly overexpressed in VCaP cells (P < 0.0001) and was also significantly elevated in PC-3 cells compared with RWPE-1 (P < 0.01) (**Figure 6C**). Furthermore, SMOC2 expression was significantly elevated in VCaP cells (P < 0.0001), whereas the difference between PC-3 cells and RWPE-1 cells was not statistically significant (P > 0.05) (**Figure 6D**). UBE4B expression was significantly higher in both VCaP and PC-3 cells compared with RWPE-1 cells (all P < 0.0001) (**Figure 6E**). These results suggest that ACACA, CROCC, RASSF3, SMOC2 and UBE4B exhibit varying degrees of abnormal expression across different prostate cancer cell lines, with VCaP cells generally showing a more pronounced trend towards high expression, suggesting that these genes may be involved in the development and progression of prostate cancer.

**Figure 6:**
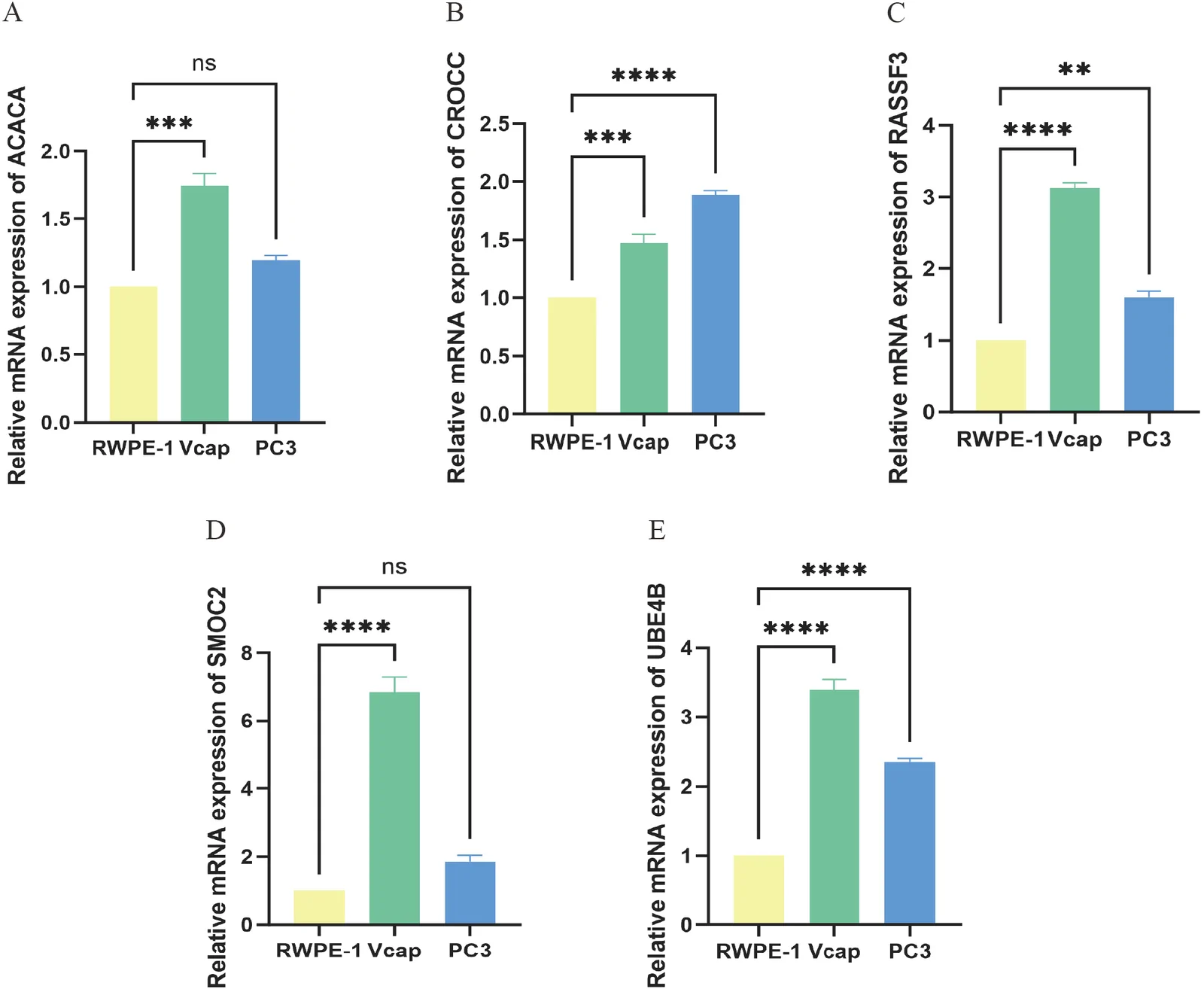
mRNA expression levels of each gene in different prostate cell lines. **(A)** ACACA was significantly highly expressed in VCaP cells but not significantly changed in PC-3 cells. **(B)** CROCC was significantly highly expressed in both VCaP and PC-3 cells, with a more significant increase in PC-3. **(C)** RASSF3 was highly expressed in VCaP cells, and also significantly increased in PC-3 cells. **(D)** SMOC2 was significantly highly expressed in VCaP cells but not in PC-3 cells. **(E)** UBE4B was significantly highly expressed in both VCaP and PC-3 cells. [Annotation: The yellow column is normal prostate epithelial cell RWPE-1. The green column is VCaP of prostate cancer cells. The blue column is prostate cancer cell PC-3. ns indicated that the difference was not statistically significant; P < 0.05, P < 0.01, P < 0.001, ****P < 0.0001.].

## 5 Discussion

The aetiology of prostate cancer has not yet been fully elucidated, but immune factors, genetic factors (Ashrafizadeh et al., 2022; Chen et al., 2023) and endocrine factors (Verhaegen et al., 2022; Wang et al., 2022; Guo et al., 2023) play significant roles in its development (Wang et al., 2022). Studies have shown that gut microbiota dysbiosis is closely associated with a variety of diseases, including obesity, diabetes, cardiovascular disease and cancer (Zhang et al., 2020; Zhang et al., 2021). The gut microbiota is a complex microecosystem comprising trillions of microorganisms, including bacteria, viruses and fungi. These microorganisms play a crucial role in maintaining host health (Qi-Xiang et al., 2022; Shuai et al., 2022; Gupta et al., 2023). Animal studies have shown that the gut microbiota diversity in rats with benign prostatic hyperplasia is significantly higher than in normal control groups (Li et al., 2022). Notably, the gut microbiota can also synthesise androgens by metabolising substances such as pregnenolone, which may accelerate the progression of castration-resistant prostate cancer (CRPC) (Pernigoni et al., 2021). Lin et al. (2024) found through comparative studies that, at the phylum level, the CRPC group exhibited a significant increase in the Firmicutes phylum, with a notable rise in the Clostridia class, whilst the Bacilli and Erysipelotrichia classes were significantly elevated in the control group. Gut microbiota are an important component of the human microecosystem. Several cutting-edge studies have demonstrated that gut microbiota play a key role in the development and progression of tumours (Zhao et al., 2023). The mechanisms involved include the direct promotion of carcinogenesis by microbiota and their metabolites; the creation of a favourable environment for tumour cell growth and spread through the regulation of cytokine secretion and signal transduction in the tumour microenvironment; and indirect carcinogenesis via pathways such as disruption of host tumour immune surveillance, promotion of inflammation, and suppression of the immune system (Zitvogel et al., 2017).

Using a two-sample Mendelian randomisation analysis, this study is the first to demonstrate, from the perspective of genetic causal inference, that the abundance of Brevibacillaceae, Brevibacillales, CAG-110 and Pararhizobium is significantly positively associated with the risk of prostate cancer (PRAD), whilst Lawsonibacter sp000492175 exerts a protective effect (**Figure 1U**). This finding is consistent with the conclusions of previous observational studies reporting that gut microbiota dysbiosis promotes the progression of prostate cancer (Liu et al., 2021), and further provides causal evidence at the genetic level (Li et al., 2026). From a biological mechanism perspective, Gram-positive and Gram-negative bacteria such as Brevibacillaceae may activate the Toll-like receptor 4 (TLR4) signalling pathway via their cell wall component lipopolysaccharide (LPS) (Li et al., 2017), inducing a chronic low-grade inflammatory response, which in turn promotes the sustained activation of the NF-κB pathway in the tumour microenvironment (Wan et al., 2016). Notably, Liss et al. observed enhanced steroid biosynthetic activity in the faeces of patients with metastatic castration-resistant prostate cancer (CRPC) (Sfanos et al., 2018; Kalinen et al., 2024; Lin et al., 2024), whilst the Brevibacillaceae and other bacterial communities identified in this study may accelerate CRPC progression by interfering with androgen metabolic pathways, consistent with previous relevant studies (Fujita et al., 2023; Huang et al., 2024). Conversely, the genus Lawsonibacter, as a potential producer of short-chain fatty acids (SCFAs), particularly butyrate, may exert a protective effect by inhibiting histone deacetylase (HDAC) activity, enhancing tumour cell apoptosis, and improving intestinal barrier function (Miya et al., 2023; Yang and Kim, 2023; Trecarten et al., 2024). Overall, the results from various meta-analyses are consistent, and the sensitivity analyses are robust, suggesting that the identified gut microbiota taxa are genetically associated with prostate cancer risk. However, the biological mechanisms underlying these associations require further experimental validation.

By combining SNP functional mapping with machine learning algorithms (Raudvere et al., 2019; Li et al., 2026), this study identified five signature genes—including RASSF3, CROCC and UBE4B—from 23 candidate genes (**Figure 2A**). Differential expression analysis revealed that FRMD3, UBE4B, RASSF3, CROCC, SMOC2 and ACACA exhibited significant differences in expression between PRAD patients and the control group; clustering heatmaps confirmed distinct intergroup expression patterns (**Figure 2A** and 2B). Chromosomal localisation analysis revealed that these six core genes are distributed across different chromosomal regions, with UBE4B and CROCC both located in adjacent regions on chromosome 1, suggesting a potential synergistic regulatory relationship (**Figure 2C**). Gene correlation analysis further revealed a significant positive correlation between RASSF3 and ACACA (Richter et al., 2009; Zhang et al., 2021), whilst SMOC2 showed a negative correlation with ACACA (Feng et al., 2022), suggesting that these genes may participate in the biological regulatory network of prostate cancer through synergistic or antagonistic interactions (**Figure 2D**). These genes represent candidate molecular features associated with microbiota-related genetic variation and PRAD susceptibility; however, their potential mediating roles require further functional validation. Among these, RASSF3 (Ras-associated structural domain family member 3), as a tumour suppressor gene, may promote cell proliferation through the activation of the RAS-RAF-MEK-ERK signalling pathway when its expression is downregulated (van der Weyden and Adams, 2007; Kudo et al., 2012; Kanno et al., 2018); whereas CROCC (Curly-root homologue) and UBE4B (ubiquitin ligase 4B) showed relatively higher expression levels in smooth muscle cell populations identified by single-cell annotation (Li and Wang, 2024), suggesting potential associations with stromal-related cellular contexts. Further functional experiments are required to determine whether these genes participate in tumour stromal remodeling or EMT regulation (Xu et al., 2020; Chaves et al., 2021). Based on the above findings, these genes may participate in the biological regulatory network of PRAD through synergistic or antagonistic interactions.

Analysis of immune infiltration revealed a close association between signature genes and the tumour immune microenvironment. Analysis based on the CIBERSORT algorithm showed significant differences between the PRAD group and the control group in the proportions of 22 types of immune cells, with statistically significant differences between the two groups observed in subpopulations such as CD4-naive T cells, follicular helper T cells and regulatory T cells (Tregs) (**Figure 3A** and 3B). Further correlation analysis (**Figure 3C**) revealed that FRMD3 was significantly positively correlated with regulatory T cells (Tregs), whilst ACACA was negatively correlated with innate immune cells such as neutrophils. The infiltration of Tregs in the prostate tumour microenvironment is typically associated with an immunosuppressive state, suggesting that high FRMD3 expression may contribute to the formation of an immune-excluded microenvironment by recruiting Tregs (Li et al., 2020; Karpisheh et al., 2021); ACACA (acetyl-CoA carboxylase α), as a rate-limiting enzyme in fatty acid synthesis, may alter the lipid metabolic profile of tumour cells when its expression is downregulated, thereby influencing the chemotaxis and activation of neutrophils (Huang et al., 2023; Liu et al., 2023). Machine learning models constructed based on these signature genes demonstrated excellent predictive performance. This study compared the predictive performance of four algorithms: GLM, SVM, RF and XGBoost. Among these, the RF model performed best, exhibiting the lowest residual distribution (**Figure 4A**) and an area under the ROC curve (AUC) of 0.94, outperforming GLM (0.90), XGB (0.90) and SVM (0.92) (**Figure 4D**). Feature importance analysis revealed that RASSF3 and CROCC are key predictive factors, ranking among the top two in terms of contribution in most models (**Figure 4B**). Calibration curve and decision curve analyses confirmed that the model exhibits good calibration and clinical net benefit (**Figures 4E, F**). A bar chart based on the five feature genes can be used for individualised risk scoring (**Figure 4G**). Analysis of the validation set showed that the model had an AUC of 0.853 (95% CI: 0.730–0.948) (**Figure 4J**), which was significantly superior to the predictive performance of individual genes (**Figures 4I, H**). This indicates that the multigene combination model can effectively distinguish PRAD patients from the general population, providing a potential molecular marker for screening high-risk individuals (Petretto et al., 2006; Brown et al., 2013; Zhang et al., 2014; Richardson et al., 2020; Rahu et al., 2025).

This study integrates single-cell RNA sequencing with spatial transcriptomics to systematically analyse cellular heterogeneity in prostate cancer tissue and the biological significance of candidate genes. With regard to cellular heterogeneity, the study identified five major cell populations: epithelial cells, fibroblasts, iPS-like cells, smooth muscle cells and tissue stem cells. The distinct distribution of these clusters suggests significant heterogeneity within prostate tissue (**Figures 5A–F**). Pseudo-temporal analysis revealed continuous transcriptional variation among annotated cell populations. Although these patterns suggest possible relationships between cellular states, they do not establish lineage differentiation or developmental trajectories (**Figure 5I**). Spatial transcriptomics further demonstrated a distinct regional distribution of different cell types: epithelial cells surround glandular-like structures, whilst smooth muscle cells and tissue stem cells are enriched in specific regions, suggesting regionalised stroma-epithelial interactions within the tumour microenvironment (Feng et al., 2024; Pakula et al., 2024; Shakeri Abroudi et al., 2025) (**Figure 5J**). Regarding candidate genes, ACACA and SMOC2 exhibited highly consistent expression patterns, both being significantly enriched in iPS-like cells and tissue stem cells (**Figures 5K–O**). As a key enzyme in fatty acid synthesis, the high expression of ACACA suggests active lipid metabolic reprogramming within the tumour microenvironment; SMOC2, as a secretory matrix protein, is enriched in the stromal regions and may be involved in extracellular matrix remodelling and tumour invasion. Their co-enrichment suggests that these genes may be associated with specific stem-like cellular populations and metabolic characteristics; however, their functional roles in regulating stemness require further experimental validation. UBE4B is primarily enriched in epithelial cells, which may reflect alterations in protein turnover under tumour conditions. FRMD3 tends to be found in tissue stem cells and epithelial cells, and may be involved in stemness maintenance; although CROCC is highly expressed in iPS-like cells, its overall expression level is low and its distribution is widespread, indicating weak specificity (Shakeri Abroudi et al., 2025; Huang et al., 2026). Spatial expression results showed that ACACA and SMOC2 signals overlapped with regions enriched for smooth muscle cells and tissue stem cell-like populations (Chen et al., 2025; Song et al., 2026). These findings indicate spatial associations between gene expression patterns and cellular localization, but do not establish their functional regulation of the tumour microenvironment. Overall, the spatial transcriptomics results corroborate the single-cell sequencing analysis, further supporting the potential roles of CROCC, FRMD3, UBE4B, ACACA and SMOC2 in regulating the prostate cancer stromal microenvironment.

The results of this study show that ACACA is significantly overexpressed in VCaP cells, whereas no significant difference was observed in PC-3 cells. This differential expression pattern may be consistent with a potential association between ACACA expression and AR signaling (Han et al., 2018). However, because AR was not experimentally manipulated in the present study, direct AR-dependent regulation of ACACA cannot be concluded and requires further validation (**Figure 6A**). CROCC was significantly overexpressed in both cancer cell lines, with a more pronounced increase in PC-3, and may promote chromosomal instability via centrosome proliferation (**Figure 6B**). RASSF3 is traditionally considered a tumour suppressor gene (Li and Wang, 2024), but was significantly overexpressed in this study; its specific role in prostate cancer requires further validation (**Figure 6C**). SMOC2 was specifically overexpressed in VCaP, possibly regulated by AR signalling and involved in tumour microenvironment remodelling (Knuuttila et al., 2014) (**Figure 6D**). UBE4B was extremely significantly overexpressed in both cell lines, suggesting that altered UBE4B expression may be associated with prostate cancer biology. However, whether UBE4B represents a therapeutic target requires further functional and pharmacological validation (Wu et al., 2011) (**Figure 6E**). Notably, ACACA and SMOC2 showed higher expression in VCaP cells, whereas CROCC and UBE4B were significantly elevated in both prostate cancer cell lines. The cell-line-specific expression patterns of ACACA and SMOC2 may be related to differences in cellular context, including AR status; however, this possibility remains speculative and requires further experimental validation.

This study has several limitations: firstly, as the GWAS data were derived from a European population, caution is required when extrapolating the findings to other ethnic groups; secondly, the gut microbiota is highly dynamic and exhibits geographical variation, and the genetic instrumental variables based on the Finnish cohort (FR02) may not fully represent the microbiota characteristics of other populations; Thirdly, although the single-cell dataset included 52 prostate tissue samples, only four benign samples were available as controls, resulting in a substantial imbalance between benign and prostate cancer samples. This limited number of benign controls may reduce the robustness of group comparisons and restrict the generalizability of the cell-type-specific findings. Therefore, the single-cell results should be interpreted with caution and further validated in larger and more balanced independent cohorts. Moreover, the limited number of biological samples available for single-cell analysis restricts the interpretation of pseudotime trajectories and cell-type-specific enrichment patterns. Although these analyses provide insights into cellular heterogeneity and gene expression distribution, they cannot independently establish cellular differentiation pathways, lineage relationships, or causal mechanisms. Future studies incorporating larger cohorts, balanced tumour and non-tumour samples, and functional validation experiments are required to confirm these findings. The generalizability of the spatial transcriptomic findings also requires validation in larger cohorts. Future research should prospectively collect data from multicentre, multiracial cohorts, combining omics approaches (metabolomics, proteomics) and organoid models to elucidate the molecular mechanisms by which specific microbial metabolites (e.g., secondary bile acids, tryptophan metabolites) regulate the tumour immune microenvironment via characteristic genes. Furthermore, the clinical value of intervention strategies targeting gut microbiota-host interactions (such as probiotic preparations and faecal microbiota transplantation) in the prevention and treatment of PRAD warrants further exploration. Additionally, this study only examined mRNA levels and lacked functional validation. Although MR provides evidence consistent with causal relationships under specific assumptions, it does not directly establish biological mechanisms. Therefore, the identified microbiota–gene relationships should be considered hypothesis-generating and require validation through functional experiments. Although SNP-to-gene mapping was applied to prioritize potential functional genes, this approach does not establish that identical causal variants drive both microbiota and PRAD associations. Future studies incorporating formal statistical colocalization approaches, such as Bayesian colocalization analysis, are required to further confirm shared causal variants and clarify the genetic mechanisms underlying the microbiota–prostate cancer relationship. Future work should expand the sample size, incorporate protein-level validation and functional experiments, and analyse their prognostic value in conjunction with clinical databases. Moreover, although repeated cross-validation and an independent validation cohort were applied during machine learning model construction, the relatively limited sample size remains an important limitation. The small cohort size may influence the stability and generalizability of the predictive model. Future studies involving larger multicenter cohorts are required to further validate the robustness and clinical applicability of the proposed gene signature. In summary, this study has revealed the abnormal expression profiles of the aforementioned genes in prostate cancer, providing experimental evidence for understanding their molecular mechanisms and developing precision treatment strategies.

## 6 Conclusion

These findings provide novel therapeutic targets and a theoretical foundation for deciphering the gut–prostate axis mechanism and advancing precision prevention and treatment strategies for PRAD. We identified four taxa, including Brevibacillaceae, Brevibacillales, CAG-110, and Pararhizobium, that were positively associated with PRAD risk, whereas Lawsonibacter sp000492175 showed a protective association. Furthermore, we identified RASSF3, CROCC, UBE4B, SMOC2, and ACACA as signature genes that are differentially expressed in PRAD and closely associated with immune microenvironment remodeling. Single-cell and spatial transcriptomic analyses revealed differential expression patterns of these genes across annotated cellular populations, suggesting potential cellular associations that require further functional validation. These findings provide potential molecular signatures and a framework for further investigation of the gut–prostate axis. Functional studies are required to determine whether these candidate genes participate directly in microbiota-related prostate cancer progression or represent clinically actionable targets.

## Data Availability

The original contributions presented in the study are included in the article/**Supplementary Material**, further inquiries can be directed to the corresponding author/s.
